# Reconstruction of functional human lips utilizing a prelaminated flap

**DOI:** 10.3389/fbioe.2024.1364705

**Published:** 2024-08-12

**Authors:** Stephen E. Feinberg, Cynthia L. Marcelo

**Affiliations:** ^1^ Emeritus in Surgery and Dentistry, University of Michigan, Ann Arbor, MI, United States; ^2^ Emeritus in Surgery, University of Michigan, Ann Arbor, MI, United States

**Keywords:** prelamination, tissue engineer, lips, reconstruction, lumen, stoma, soft tissue injuries, function

## Abstract

Lips form a structure that are difficult to reconstruct after a traumatic avulsion injury or cancer ablative surgery secondary to loss of volumetric muscle mass. Traditional tissue engineering approaches of *in vitro* fabrication of mature tissue constructs can supply an alternative to the current surgical standard of care for functional lip reconstruction. We demonstrate a hybrid approach that combines the advantages of *in situ* muscle flap prefabrication with *in vitro* fabrication of an autogenous mucocutaneous construct as the laminate for prelamination to form a designer microvascular muscle free flap for lip reconstruction.

## 1 Introduction

Lips form a structure that are difficult to reconstruct after a traumatic avulsion injury or cancer ablative surgery secondary to loss of volumetric muscle mass because they represent a dynamic composite tissue of mucosa, skin, and innervated skeletal muscles. Significant loss of lips is a functional and esthetic concern because the neuromuscular control of normal lip structures is required for eating, drinking, talking and social gesturing. Traumatic avulsion of the lips or loss secondary to cancer ablative surgery is a survivable injury; however, without functional lip reconstruction (restoration of muscle volume and reinnervation of the end organ) life for affected individuals is burdened by drooling, food spillage while eating, unintelligible speech, social rejection and is emotionally devastating. The “Gold Standard” for lip reconstruction is a composite free radial forearm-palmaris longus tendon flap. This results in microstomia and an inanimate “stiff” non-functional lip. Microstomia is a common morbidity of substandard lip reconstruction, a consequence of inadequate tissue bulk (muscle volume), which impairs denture utilization and interferes with adequate nutritional uptake and speech. Functional reconstruction of lips is critical when > 50% of the lips are lost. Alternative treatments such as autogenous tissue flap reconstruction or allogeneic face transplantation are inadequate.

Tissue engineering and regenerative medicine (TE/RM) is a viable alternative but face several barriers: 1) inability to create functional composite soft tissue structures (striated muscle, skin, and mucosa) and 2) difficulty developing an *in vivo* perfusion system (blood vessels) to supply nutrition for large segments of tissue created *in vitro*. We propose a novel approach to restore muscle volume and lip function utilizing a prelaminated innervated prevascularized prefabricated (PIPP) microvascular free flap based on the latissimus dorsi muscle (LDM). The laminate is a mucocutaneous construct (MCC) composed of autogenous primary oral and skin keratinocytes obviating the need for lifetime immunosuppression seen with allogeneic grafts. The PIPP flap addresses barriers preventing a TE/RM approach: tissue complexity and vascularity as well as restoring soft tissue volume (muscle) and motor reinnervation to the lips.

None of them, however, present a tissue composite with a M/C junction. For this reason, development of continuous human oral mucosa-lip-skin constructs or with other M/C junctions is necessary. The oral mucosa forms the inner aspect of the lip and extends and unites with the skin by a junction known as the vermillion border that unites the oral mucosa with the skin of the face (Binnie and Lehner, 1970; Zugeman, 1986).

## 2 Materials and methods

### 2.1 Manufacture of a mucocutaneous construct for prelamination

We used previously frozen oral and skin adult human keratinocytes to fabricate a composite laminate. Full thickness human skin (from a breast reduction) and oral mucosa explants (taken from the upper inner aspect of the lip) were obtained from discarded material from surgeries performed at the University of Michigan Health System. The Institutional Review Board approved use of the mucosa and skin, and donors provided informed consent for research use. Preparation of the explants and extraction of keratinocytes was performed as previously reported by Marcelo (Marcelo et al., 1978) for skin and by Izumi et al. (2004); Izumi et al. (2000) in the case of oral mucosa. Tissue samples were digested with a 0.04% trypsin solution (oral tissues) or 0.125% trypsin solution (skin tissues) overnight at room temperature (Sigma-Aldrich, St Louis, MO). Enzymatically dissociated keratinocytes were plated at a density of 7.0 · 10^6^ cells per T-25 flask (Corning, Corning, NY). The cells were cultured without a feeder layer, fetal bovine serum, or bovine pituitary extract in an animal product-free culture medium, EpiLife M-EPI-500, (Cascade Biologics, Portland, OR) that was supplemented with EpiLife defined Growth Supplement (EDGS; Cascade Biologics). In addition, the medium was supplemented with 25 mg/mL of Gentamicin (Sigma-Aldrich) and 0.375 mg/mL of Amphothericin B (Gibco, Invitrogen Carlsbad, CA). Primary keratinocytes were harvested when 70%–80% confluent and were subsequently subcultured for use in the fabrication of the laminate. In these techniques, both oral and skin keratinocytes are expanded in a serum-free defined low calcium medium (0.06 mM CaCl2). The primary oral and skin keratinocytes were cultured and grown in separate culture flasks and passaged to reach passage two and then frozen. Afterward, they were thawed and cultured to reach the necessary number of cells for seeding on the dermal allograft, at a density of 2.5 × 10^5^ cells/cm^2^. This portion of the manufacturing protocol can vary in time and is mostly dependent on the need for oral keratinocyte amplification given that the oral explants are typically smaller. The number of keratinocytes necessary for fabrication of the 3D lip construct must be calculated because it is a function of the size of the surgical site grafting. The AlloDerm^®^ was presoaked in 5 mg/cm2 human type IV collagen (Becton Dickinson Labware, Bedford, MA) for a minimum of 3 h before keratinocyte seeding. Once enough of both skin and oral keratinocytes had been grown in culture flasks, they were seeded in two separate compartments on the same piece of type IV collagen coated AlloDerm^®^ utilizing our patented custom culture ware that allows us to create an interface between the two keratinocyte populations (Peramo et al., 2012). The process consists of growing human oral and skin keratinocytes into a three-dimensional (3D) construct to form a M/C junction containing stratified oral mucosa and stratified skin on the same dermal component.

**FIGURE 1:**
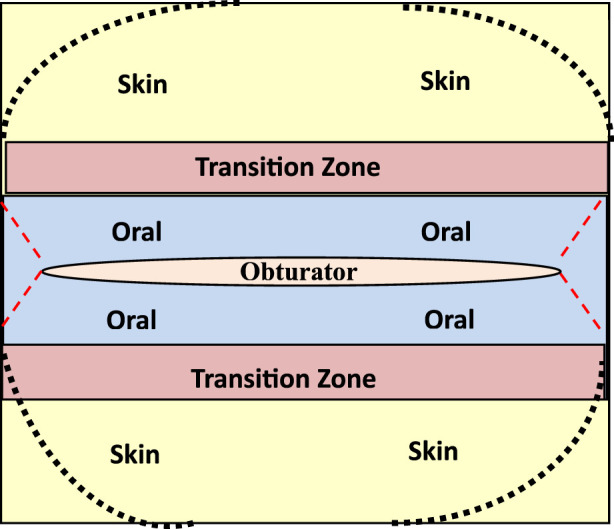
Template to fabricate muco-cutaneous constructs illustrating distinct zones for seeding of skin and oral keratinocytes with a transitional zone that is created with a physical barrier that is removed from the laminate once it is grown at an air-liquid interface. Note an opening in the template for placement of an obturator to create a patent stoma.

Once both oral and skin keratinocytes the cells are seeded over an Alloderm^®^ membrane into separate compartments they are temporarily divided (overnight) by a removable barrier made of a biocompatible material such as silicone. After several days in culture the whole construct is raised to the air/liquid interface to become a 3D organotypic construct with a transitional zone noted between the skin and oral keratinocytes. Phenotypic markers specific for skin and oral keratinocytes showed three distinct areas on the organotypic construct like the pattern one sees *in situ* for lip epithelium (Binnie and Lehner, 1970; Barrett et al., 2005). We have designed a patented barrier apparatus to assist in the manufacture of our M/C laminate (Feinberg and Roger, 2022) using the template shown in Figure 1.

### 2.2 Development of an animal model

We developed an athymic rat animal model to obtain data to show the efficacy of our novel, tissue engineered medical product (TEMP) and obturator for incorporation into a prelaminated, innervated, prevascularized, prefabricated (PIPP) microvascular free flap for use in dynamic lip reconstruction. The University of Michigan Committee on Use and Care of Animals approved all animal studies.

**FIGURE 2:**
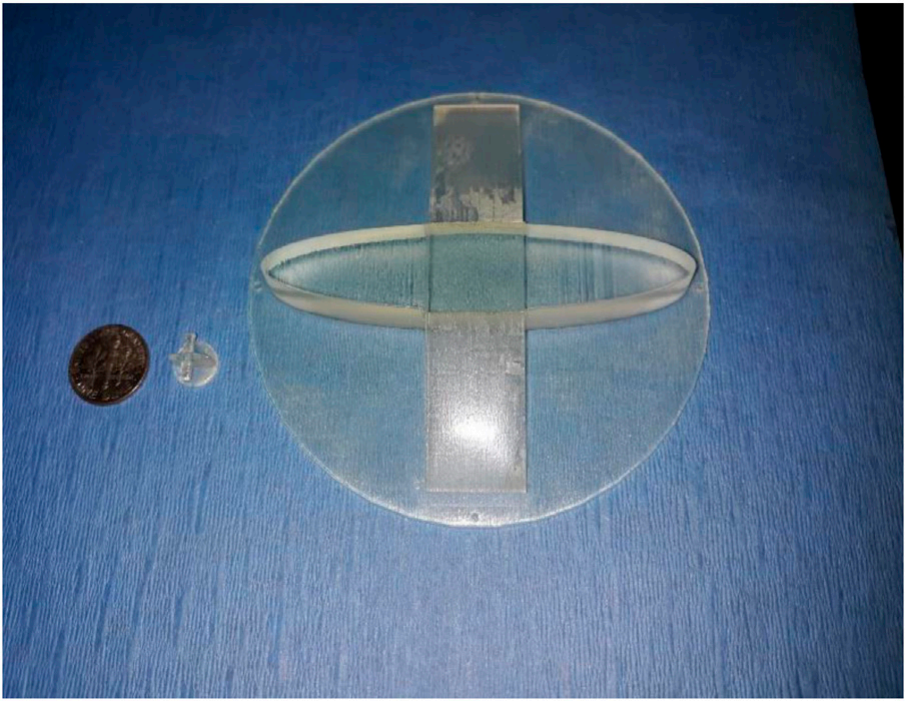
Obturator design for both the animal (rat) and human obturator. Note differences in size in relationship to a standard USA coin: dime.

**FIGURE 3:**
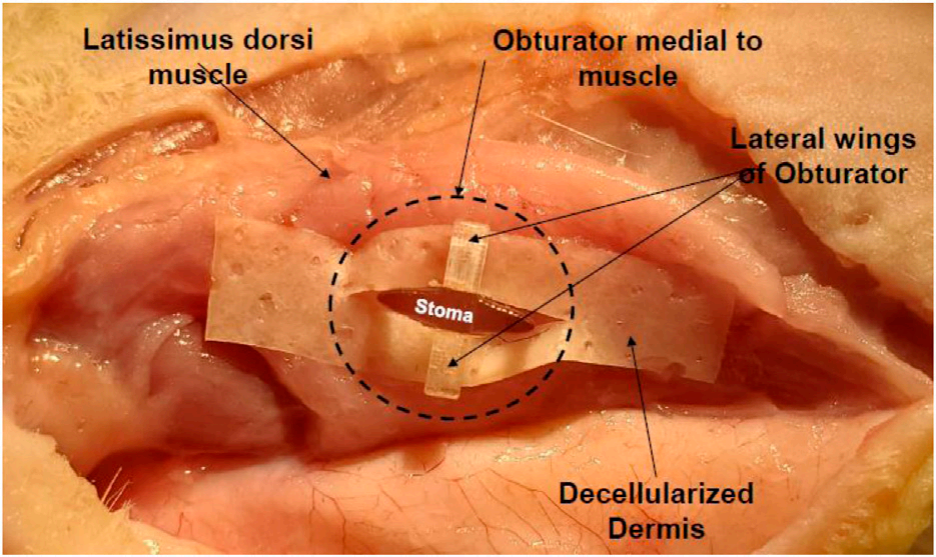
Obturator *in situ* in a cadaveric rat illustrating its placement and design to create a patent stoma in relation to the latissimus dorsi muscles. The laminate (decellularized dermis) contains no cells.

Once the mucocutaneous construct (MCC) or TEMP was fabricated it was implanted onto the immunodeficient rats via the following procedure:

Rats were anesthetized via isoflurane inhalation and surgically prepped. The latissimus dorsi muscle (LDM) was then isolated through a 6 cm incision from head to tail and dissected free of the underlying connective tissue with preservation of the neurovascular bundle nerve to preserve the viability and innervation of the muscle. At this time the M/C constructs were grafted onto the muscle bed and a circular piece of gas sterilized biomedical-grade silicone sheeting, 0.005 in thick was placed above and below the grafts to prevent adherence of the epithelial layers of the tissue composite to the connective tissue of the subcutaneous pouch. The open reticular portion of the M/C constructs was grafted onto the muscular fascia and sutured in place to allow integration of the laminate and formation of a microvascular network. The TEMP or MCC was then implanted onto the latissimus dorsi muscle (LDM) with a novel and patented silicone obturator (Feinberg et al., 2020) (Figure 2) placed between the muscle fibers and MCC to maintain the stoma patency during the 2-week *in vivo* lamination period (Figure 3). After 2 weeks, the obturator was removed and the contractile capacity of the integrated MCC and stoma patency was evaluated by stimulation of the thoracodorsal motor nerve. Rats were then euthanized, and tissues were harvested to assess vascularity and integration of the MCC. TEMP maturation was assessed with histology and immunohistochemistry (IHC) staining to define areas of continued existence of skin and mucosal keratinocytes on the TEMP. Antibodies used were: Specific for oral mucosa: SPRR3, rabbit polyclonal react with human (Sigma-Aldrich, HPA044467) and Specific for skin: K2 (cytokeratin), mouse monoclonal react with human (Progen Biotechnik, 65191).

## 3 Results

**FIGURE 4:**
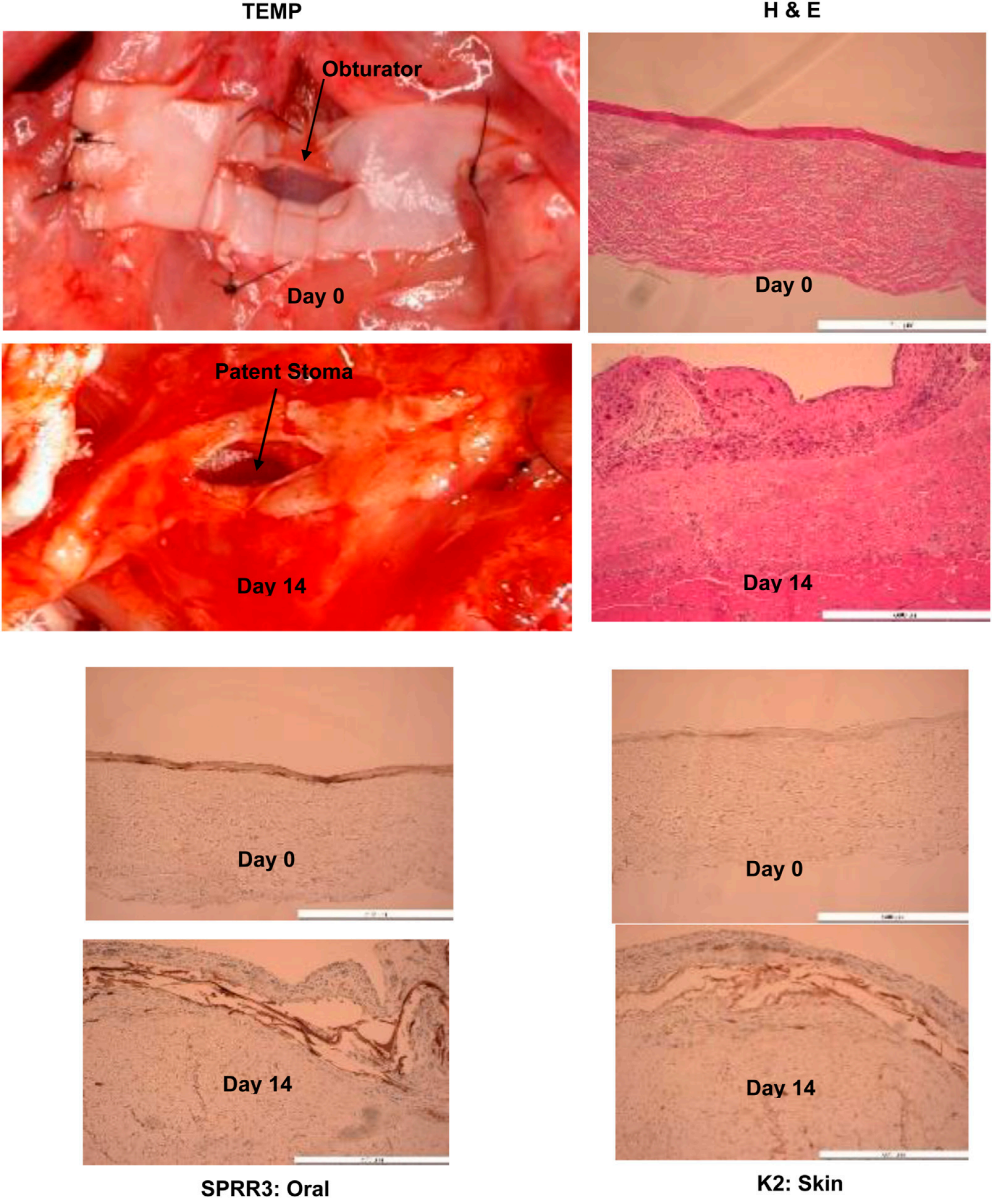
Histology, H & E and immunohistochemistry (SPRR3: Oral and K2: Skin) showing evidence of tissue engineered medical product’s (TEMP) integration with latissimus dorsi muscle, presence of oral and skin keratinocyte survival in concert vascularization of the tissue engineered medical product laminate at Day 14 (note change in color) and stomal patency after removal of obturator. Obturator stomal patency and muscle function can be seen at this link:www-personal.umich.edu/∼jbwash/082721Rat2Video_crop.mp4. Antibodies used were: Specific for oral mucosa: SPRR3, rabbit polyclonal react with human (Sigma-Aldrich, HPA044467). Specific for skin: K2 (cytokeratin), mouse monoclonal react with human (Progen Biotechnik, 65191).

Six rats underwent the surgical protocol with 100% survival rate. Seroma formation was observed in three rats which required serial aspiration and led to modification of the surgical technique for the subsequent rats with addition of a Penrose drain and pressure dressing at the time of TEMP implantation. There was no evidence of infection or wound breakdown during the 2-week implantation period. Contractile capacity of the LDM via stimulation of the thoracodorsal nerve showed a patent stoma in all rats with maintenance of the lumen size. Histology confirmed evidence of vascular perfusion via microcapillary formation via inosculation and integration of the TEMP (MCC) laminate to the underlying muscle (Figure 4). Persistent zones of oral mucosa and skin epithelium were verified by IHC staining for SPRR3 and K2 respectively (Figure 4).

## 4 Discussion

Lip reconstructions are limited by locally available, non-scarred and viable tissue. The radial forearm flap used to reconstruct lips is the primary alternative approach involving a fasciocutaneous flap which has major deficiencies (Lew et al., 2010). It does not consist of muscle and therefore is not a viable alternative for a prelaminated flap for lip reconstruction. Patients treated with a radial forearm flap have a stiff lip with no evidence of animation or innervation. The most morbid potential complication of lip reconstruction is microstomia, which is a consequence of inadequate tissue bulk (muscle volume). This may impair denture utilization and, at worst, interfere with adequate nutritional uptake and speech. This is a common occurrence that results from contemporary lip reconstructive techniques due to a shortage of available well-perfused composite soft tissue that we can address with our proposed designer PIPP free flap transfer to the lips. Flap prefabrication allows the reconstructive surgeon to choose an uninjured wound bed with adequate soft tissue characteristics, an appropriate set of vessels, a motor nerve, and a place to fabricate the construct.

To overcome the limitation in surgical options to restore functional reconstruction of the lips, we use the surgical technique of prelamination to create a designer (customized) prelaminated innervated prevascularized prefabricated (PIPP) composite soft tissue microvascular free flap based on the latissimus dorsi muscle (LDM) to develop the “next-generation” soft tissue implant for complex soft tissue reconstructions. Lamination refers to the process of bonding of layers. Prelamination designates a reconstructive process whereby a three-dimensional (3D) structure is built at a remote site by laminating different layers of components as composite grafts into a reliable existing axial vascular bed. This is done in close association to a motor nerve, allowing the structure 2 weeks to mature before transferring the unit to the defect based on its native axial blood supply and motor innervation. The technique of prelamination allows reconstruction to begin at a remote site. This is important because the recipient site being reconstructed may lack the blood supply or healthy tissue necessary to support construction of a sophisticated 3D construct at the defect site. Remote reconstruction in an unscarred vascular bed offers the best chance for the composite grafts to mature (Pribaz et al., 1999). Prelamination is often used in reconstructing structures with multiple functional layers, i.e., full-thickness reconstruction of nose, lip, cheek, ear, maxilla, mandible, and trachea, but still lacks the unique design of our customized PIPP microvascular flap (Vranckx et al., 2005; Vranckx, 2006). This allows us to use the body as an *in situ* bioreactor. A distinctive feature of *in situ* tissue engineering is that any engineered constructs that are implanted are not functionally mature and on implantation continue to differentiate and mature (Sengupta et al., 2014).

Our Stomatogenesis™ hybrid approach combines the advantages of *in situ* muscle flap prefabrication with *in vitro* fabrication of a MCC as the laminate for prelamination. The tissue engineered primary human oral mucosa and skin keratinocytes on an acellular dermal matrix (ADM), our mucocutaneous constructs, are cultured *in vitro* and transferred onto an existing muscle, such as the LDM, which can be harvested as a PIPP flap. In a defined period, the MCC matures, integrates with the muscle, and develops a microcapillary system perfused through a process of inosculation from the major artery and vein of the LDM. The PIPP flap is then transferred, after removal of the obturator, to the area of the lips to restore soft tissue muscle volume, mucosa, skin, striated muscle, and motor reinnervation to the lips upon anastomosis of the thoracodorsal artery, vein, and the motor nerve to the facial nerve.

In the procedure, vascular and nerve pedicles are micro-anastomosed to existing pedicles in the face. In all lip reconstructive cases, a full lip mucocutaneous construct with a functional stoma will be created, which will be fabricated based on the lip template in Figure 2 and implanted onto the PIPP flap as noted in Figure 1. This will address the size and geometry of the variation of defects to be reconstructed. It is easier to modify the PIPP flap at the time of harvest than to create a variety of different shaped lip constructs. The MCC will be created in a cGMP facility or closed system bioreactor. The stoma in the MCC laminate will be maintained by our novel patented obturator (Figure 2).

There are several advantages of combining *in vitro* construction of lip epithelium and ADM with an *in-situ* bioreactor approach (i.e., using the body to assist in device fabrication after implantation) for creating a designer PIPP flap to reconstruct craniofacial soft tissue injuries:

1) Address one of the primary impediments in TE/RM by creating immediate vascularity and perfusion and motor innervation of the construct through surgical microvascular anastomosis of the muscle’s neurovascular bundle to the facial vessels/nerves.
2) Create a composite soft tissue graft composed of epithelium (oral and skin), dermis and muscle, *in situ* with the LDM, to increase soft tissue volume (bulk) that is lacking at the recipient site (lips).
3) Decrease in donor site morbidity because we will avoid using extremities as a tissue donor site, thus shortening the rehabilitation phase.
4) Restoration of function will be faster and the outcomes better, including:
  a. A decrease in the number of surgeries needed with a concomitant decrease in operating room time.
  b. Enhancement in the quality, shape and function of the soft tissue being regenerated.
  c. Inclusion of a motor nerve to restore function.
5) The ultimate source of the cells to develop the soft tissue constructs (lips) will come from the patient. Small punch biopsies will be excised from non-keratinized oral mucosa and the posterior auricular region for skin (optimal color match to face), making the construct autochthonous (self to self). This will circumvent the need for immunosuppression. Color match can be modified or fine-tuned by tattooing (Fernandes and Clemow, 2012).

An important advantage of the technique presented is the fact that the 3D lip constructs are manufactured in serum free medium modified to contain totally defined animal-free supplements and without the use of feeder layers (Izumi et al., 2000). This culturing approach will allow grafting the 3D lip constructs, back into autogenous human recipients with minimal risk of cellular cross-contamination or immunological rejection (Izumi et al., 2011). The approach presented in tissue engineering 3D lips will have several advantages for the reconstruction of craniofacial soft tissue injuries. In particular, the source of the cells to develop the 3D skin lips constructs will come from small punch biopsies from the oral mucosa and skin of the patient, thus making the construct autochthonous.

The most important goal of lip reconstruction is to restore function that is dependent on oral competence and size of the oral aperture. To accomplish this, we must maintain oral circumference with a viable, well-perfused microvascular flap with sufficient bulk to perform the necessary surgical repairs. This requires restoring continuity of the labial vestibule and orbicularis oris muscle (restoration of striated muscle volume), a major striated muscle of the lips, with an intact motor innervation. The best results occur with a completely intact sphincter with active motor function and sensory sensation.

We have successfully developed an animal model using a TEMP integrated to the LDM with incorporation of an obturator to maintain stoma patency. The athymic rat model showed that it is possible to develop a prelaminated, innervated, prevascularized, prefabricated microvascular free flap for dynamic, functional reconstruction of complex, composite soft tissue defects of the lip.

## Data Availability

The raw data supporting the conclusions of this article will be made available by the author, without undue reservation.
